# Recent advances in the management of chronic obstructive pulmonary disease

**DOI:** 10.12688/f1000research.9819.1

**Published:** 2017-06-09

**Authors:** Sharon R Rosenberg, Ravi Kalhan

**Affiliations:** 1 Asthma and COPD Program, Northwestern University Feinberg School of Medicine, Chicago, IL, UK

**Keywords:** COPD, LAMA, LABA, ICS

## Abstract

Novel pharmacotherapies introduce additional options to providers and patients in how to best treat chronic obstructive pulmonary disease (COPD). Emerging data question the role of inhaled corticosteroids in COPD treatment, particularly as combination dual bronchodilator pharmacotherapies demonstrate robust results. For those maximized on pharmacotherapy with continued dyspnea or exacerbations or both, emerging bronchoscopic procedures may offer additional therapy in select patients. This review focuses on data supporting the use of novel ultra bronchodilators, particularly in combination, and on the role for inhaled corticosteroid withdrawal and new bronchoscopic procedures.

## Introduction

Chronic obstructive pulmonary disease (COPD) is projected to become the third most common cause of death worldwide by 2030 ^1– 3^. Acute exacerbations of COPD are associated with worsening symptoms, including breathlessness, decreased quality of life (QOL) ^4^, and an accelerated loss of lung function ^5^. Those hospitalized for acute exacerbations of COPD are at an increased risk of one-year mortality of at least 18% ^6^. The majority of an estimated $50 billion cost associated with COPD care in the United States is spent treating acute exacerbations ^7^. An array of emerging pharmacotherapies challenges the traditional way COPD has been managed. This review will focus on the current evidence for use of combined long-acting muscarinic antagonists (LAMAs) with long-acting beta-2 agonists (LABAs), withdrawal of inhaled corticosteroids (ICSs), and emerging data on bronchoscopic interventions in COPD.

## Ultra long-acting beta-2 agonists

Long-acting bronchodilators improve lung function, thereby improving symptoms and exercise performance, and prevent exacerbations ^8– 10^. Long-acting bronchodilators show similar efficacy in patients with moderate compared with more severe COPD ^10, 11^, indicating that forced expiratory volume in one second (FEV _1_) does not predict bronchodilator treatment response. Several once-daily LABAs have become available over the past several years, and indacaterol, olodaterol, and vilanterol are the newest. The existing drug classes (beta-2 agonists and muscarinic receptor antagonists) work by relaxing airway smooth muscle tone, leading to reduced respiratory muscle activity and subsequent reduction in airway resistance and making it easier for patients to breathe. Bronchodilation aims at alleviating bronchial obstruction and airflow limitation, reducing hyperinflation, improving emptying of the lung and exercise performance ^12, 13^, thus improving dyspnea. This explains why all current COPD practice recommendations highlight that inhaled bronchodilators are the mainstay of current management regardless of disease severity ^14– 16^.

**Figure 1.**
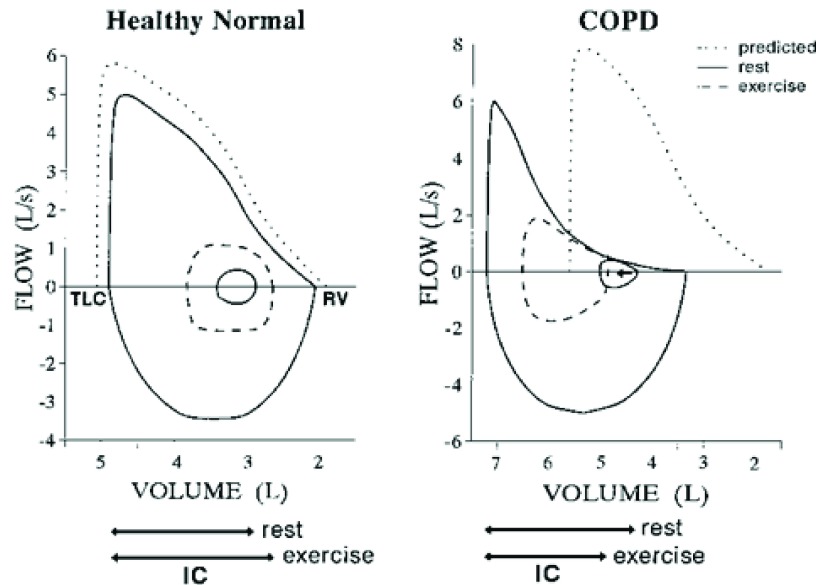
Schematic representation of a normal subject (left) and dynamic hyperinflation in a chronic obstructive pulmonary disease (COPD) subject (right) at rest and during exercise. IC, inspiratory capacity; RV, right ventricle; TLC, total lung capacity.

Hyperinflation is a common occurrence leading to breathlessness in COPD. Lung volumes are stable when the tidal volume is completely exhaled prior to the next breath. As the tidal volume increases with exercise, expiratory muscles are recruited to increase pleural and alveolar pressures and increase expiratory flow to ensure that the increased tidal volume is completely exhaled. Hyperinflation occurs when the end-expiratory volume is increased, typically because of airflow limitation, such as in COPD. Compared with healthy patients, patients with COPD have decreased elastic recoil pressure such that the elastic recoil pressure falls to zero at a larger end-expiratory volume. Hyperinflation may also occur as the airways in patients with COPD collapse when the pleural pressure is positive, preventing increased expiratory flow ^17, 18^, and therefore exhalation may not be completed prior to the onset of the next breath ^19, 20^ ( Figure 1).

The efficacy of ultra LABAs is well established. Among others, two randomized, double-blind, placebo-controlled, parallel-group phase 3 studies have shown the long-term efficacy and safety of once-daily olodaterol 5 and 10 μg in patients with moderate to severe COPD continuing usual-care maintenance therapy ^21^. Lung function effects of indacaterol are significantly greater than those of the traditional (twice-daily dosing) LABAs formoterol, salmeterol, and arformoterol and are similar to those of the LAMA tiotropium ^22– 26^.

The debate as to which class of inhaled bronchodilator should be the first-line agent in COPD continues. Guidelines do not distinguish which long-acting bronchodilator agent, LABA or LAMA, should be considered first line, but rather only that the use of a long-acting bronchodilator agent is advised ^14, 15^. Although a randomized, placebo-controlled 6-month trial of tiotropium versus salmeterol was conducted ^27^, it is debatable whether such a direct LABA-versus-LAMA comparison will be performed, particularly given the increasing number of options available ^28^.

Cautious indirect comparisons may be made from the existing clinical trial database about how each class of drug impacts COPD outcomes, although the limitations of this approach are obvious. Nonetheless, limitations aside, LABAs are more effective than LAMAs if we consider symptoms or health-related quality of life (HRQOL) as the primary outcome ^29^, although LAMAs also impact favorably on both outcomes ^28^. By contrast, LAMAs appear to be more effective than LABAs if exacerbations are the expected primary outcome, regardless of whether LABAs are administered on a twice-daily ^30^ or once-daily ^31^ basis. The outcome of interest may largely determine which bronchodilator to start with in a patient with COPD ^32^. In the symptomatic patient, there is no substantial difference between LABAs or LAMAs, whereas in frequent exacerbators it seems preferable to use a LAMA.

## Long-acting muscarinic antagonists

Until recently, tiotropium was the only globally available ultra LAMA, and it has a rich database of efficacy outcomes in COPD ^3, 9, 33^. Over the past few years, data have emerged documenting the efficacy of other drugs in the LAMA class ( Table 1). The ACCLAIM trials documented an improvement in FEV _1_ and delayed time to first exacerbation with once-daily aclidinium treatment ^34^. Further study with twice-daily aclidinium in the ATTAIN trial showed significant increase in trough and peak FEV _1_, dyspnea, and improvement in quality of life (QOL) scores ^35^. Umeclidinium significantly improved trough FEV _1_, dyspnea, and QOL scores ^36^. The GEM (Glycopyrrolate Effect on Symptoms and Lung Function) 1 and 2 studies of glycopyrronium versus placebo show improvements in FEV _1_, dyspnea, QOL scores, and rescue medication use in patients with moderate to severe airflow limitation ^37^. In recent studies of a novel soluble glycopyrrolate solution delivered via the investigational eFlow ^®^ nebulizer, the nebulized LAMA formulation was reported to be safe and well tolerated, and there were no significant changes in cardiovascular signs and electrocardiography parameters ^38^. There was a dose-related and clinically significant improvement in FEV _1_ following nebulized glycopyrrolate, providing support for its development as a convenient nebulized LAMA bronchodilator for patients with COPD ^38^. US Food and Drug Administration (FDA) approval would bring forth a novel nebulized ultra LAMA option available to patients with COPD. Availability of a nebulized LAMA would greatly complement the currently available nebulized LABA medications (formoterol and arformoterol). Of 400 caregivers and patients with COPD randomly surveyed via phone, the overwhelming majority were satisfied with traditional nebulization therapy, reporting benefits in symptom relief, ease of use, and improved QOL ^39^.

**Table 1.** Key findings of recent LAMA and dual-agent LAMA/LABA trials reviewed.

Trial	Pharmacotherapy	Results
Jones *et al*. ^34^ (2011) Jones *et al*. ^35^ (2012)	Aclidinium	Improved FEV _1_, delay to first exacerbation
Trivedi *et al*. ^36^ (2014)	Umeclidinium	Improved FEV _1_, dyspnea, QOL
LaForce *et al*. ^37^ (2016)	Glycopyrronium	Improved dyspnea, QOL
Wedzicha *et al*. ^32^ (2016)	Glycopyrronium/indacaterol versus salmeterol/fluticasone	Decreased exacerbations
Singh *et al*. ^47^ (2014) D’Urzo *et al*. ^48^ (2014) Bateman *et al*. ^49^ (2015)	Aclidinium/formoterol	Improved dyspnea and exacerbations, delay to first exacerbation
Buhl *et al*. ^51^ (2015)	Tiotropium/olodaterol	Improved FEV _1_, QOL
Donohue *et al*. ^45^ (2014)	Umeclidinium/vilaterol	Decreased exacerbations

FEV _1_, forced expiratory volume in one second; LABA, long-acting beta-2 agonist; LAMA, long-acting muscarinic antagonist; QOL, quality of life.

## Dual-agent long-acting bronchodilators

For patients with COPD whose disease is not well controlled—whether in terms of symptoms or exacerbation frequency as recommended by the Global Initiative for Chronic Obstructive Lung Disease (GOLD) COPD statement—with a single long-acting bronchodilator, the most recent guidelines depart from prior use of ICSs and recommend the use of dual long-acting bronchodilators, unless a recurrent or severe exacerbator ^14^. Studies showing benefit of combination LABA and LAMA in separate devices with both short- and long-acting components ^40– 42^ prompted the development of a single component with multiple long-acting bronchodilators. The first of the ultra LAMA/LABA combination inhalers approved was for umeclidinium/vilanterol. Umeclidinium/vilanterol appears to be safe, produces greater improvements in lung function compared with monocomponents, and in some studies reduces the risk of exacerbations ^43– 46^. However, umeclidinium/vilanterol combination, compared with tiotropium or the monocomponents umeclidinium or vilanterol, has not shown dramatic improvements in dyspnea or HRQOL. The bulk of the data for the aclidinium/formoterol combination is from two 24-week randomized, placebo-controlled studies—AUGMENT and ACLIFORM studies—showing improved lung function, dyspnea, and HRQOL ^47, 48^. Combining data from these two studies showed reduced exacerbations ^49^ and similar cardiovascular events over 6 months for the twice-daily-administered LAMA/LABA compared with placebo ^50^.

The FDA approved tiotropium/olodaterol in a soft mist inhaler in 2015. In a 6-week crossover study, it improved lung function, and a combined analysis of the TOnado 1 and 2 studies documented improved dyspnea and HRQOL ^51^. These studies were not powered for exacerbation reduction, but other LAMA/LABA combinations have been shown to reproducibly reduce exacerbations. In a multicenter trial, glycopyrronium/indacaterol once daily was compared with fluticasone/salmeterol twice daily in 3,362 patients with moderate to severe COPD with a history of at least one moderate to severe exacerbation in the previous year ^52^. Glycopyrronium/indacaterol reduced the rate of mild to severe COPD exacerbations by 11% compared with fluticasone/salmeterol over the 52-week trial ^52^. Patients with a history of two or more moderate exacerbations or one hospitalization in the previous year had similar exacerbation rates between the two treatment arms. Of note, glycopyrronium/indacaterol was associated with slightly fewer episodes of pneumonia (3.2%) compared with fluticasone/salmeterol (4.8%). The combination of a long-acting anticholinergic plus an inhaled glucocorticoid has not been compared with a long-acting anticholinergic alone.

Patients with dyspnea despite the use of either a LAMA or LABA may have improvement in HRQOL, dyspnea, and reduced rescue medication use with LAMA/LABA combination, and some agents have reported improvement in exacerbations. However, the degree of symptom improvement along with lung function improvement remains a clinical question of extreme importance. Early studies of LAMA/LABA combination describe transition dyspnea index and Saint George’s Respiratory Questionnaire meeting the minimal clinically important difference (MCID) for breathlessness versus placebo whereas individual monocomponents did not ^43, 47, 53, 54^. Of note, the primary outcome of these studies was lung function, not patient-reported outcomes (PROs). Statistically significant differences have been shown for subsequent studies with PROs as the primary endpoint ^55, 56^ as well as a pooled analysis ^49^. They provide a signal but are below the MCID thresholds. An associated reduction in reliever medication use suggests clinical relevance ^54, 55^, although overall the clinical impact of LAMA/LABA combination versus its monocomponents is unclear. Evidence of excessive ICS/LABA prescribing, coupled with emerging data (discussed below) of safe steroid withdrawal, makes LAMA/LABA combination more reasonable, particularly with the aim of maximizing bronchodilation in those with persistent dyspnea.

## A role for steroid withdrawal

As new clinical trial data emerge, a debate regarding the appropriate role of ICS withdrawal in COPD has formed. Several earlier studies reported that an abrupt withdrawal of ICS precipitates exacerbations and results in a deterioration in lung function and symptoms ^57– 59^. There has remained equipoise around this issue, however, and a meta-analysis of three of these older trials, the only trials deemed to be acceptable in terms of quality and level of bias, determined that withdrawal of ICS was not actually associated with any statistically significant increase in the exacerbation rate and that the effects on other outcomes, such as lung function and health status, were inconclusive ^60^. Methodological limitations marred these studies, and the contradictory findings may be due to differences in heterogeneity in patient characteristics, disease severity, and outcome definitions among other factors ^57– 60^.

Recently, two randomized controlled trials and a prospective study revealed that ICS can be safely withdrawn in certain patients. Those with COPD and a low risk of exacerbations should not be prescribed ICS-containing regimens, according to the latest (2017) GOLD guidelines ^14^. However, a large proportion of patients are already initiated on ICS-containing regimens ^5, 61– 63^. Recent studies evaluating GOLD groups A and B (individuals with relatively preserved lung function and not at risk for exacerbations but perhaps for high burden of symptoms) have shown no consequences associated with ICS withdrawal. It was prospectively demonstrated that withdrawal of ICS in patients with symptomatic, moderate COPD with fewer than two exacerbations a year was not associated with any deterioration in lung function, symptoms, and exacerbation rate over a 6-month observation period ^64^.

A subsequent randomized controlled trial of those with moderate COPD and no prior exacerbation history found that switching from a fixed-dose combination of ICS/LABA to a LABA was not associated with any differences in lung function, symptoms, health status, and exacerbations ^65^. These studies support the current GOLD recommendations that groups A and B do not benefit from ICS-containing regimens. Furthermore, they suggest that ICS therapy can be safely withdrawn from patients with moderate COPD and a low risk of exacerbations who continue taking long-acting bronchodilators. A growing armamentarium of novel, ultra LABA, ultra LAMA, and LABA/LAMA combinations can be considered to optimize bronchodilation and permit ICS withdrawal.

The WISDOM trial was the largest and first to examine stepwise withdrawal of ICS in patients with COPD receiving maintenance therapy of long-acting bronchodilators, including those at risk for exacerbations. The stepwise withdrawal of glucocorticoids was non-inferior to the continuation of such therapy with respect to the risk of moderate or severe exacerbations ^66^. The WISDOM trial findings indicate that not all patients benefit from including ICS in their treatment regimen despite current guidelines. Whether a subset of patients will benefit from continuing an ICS-containing regimen and how to identify such a population has not been studied, although subgroup analyses of the WISDOM trial did not show differences in exacerbation occurrence with respect to age, sex, smoking status, body mass index, ICS or beta-blocker therapy at screening, chronic bronchitis, GOLD stage and group, and prior therapy with antibiotics or systemic glucocorticoids ^66^. This has led to our practice of withdrawing ICS in patients with stable COPD. This practice is further supported by a recent landmark study where a combination LAMA/LABA was superior to a combination ICS/LABA in preventing exacerbations ^52^.

## Emerging bronchoscopic therapies

Lung volume reduction surgery is the only surgical procedure to prolong life in COPD ^67^. However, only a particular subset of patients, those with known upper lobe predominant emphysema and low post-rehabilitation exercise capacity, derive a mortality benefit. The opportunity to expand the population with severe emphysema who may benefit from intervention by less invasive means has driven the development of potential bronchoscopic interventions for severe emphysema. A recent trial evaluated the efficacy, safety, cost, and cost-effectiveness of nitinol coils versus usual care in patients with severe emphysema ^68^. The trial randomly assigned patients to a usual-care arm consisting of rehabilitation and bronchodilators with or without ICS and oxygen or to the treatment arm where patients received usual care plus additional therapy of approximately 10 coils per lobe placed in two bilateral lobes in two procedures. The study resulted in improved exercise capacity with high short-term costs. A second study of lung volume reduction coils showed a wide range of clinical outcomes among study participants, and some experienced important improvements in exercise tolerance and lung function whereas others had a less robust result. Although the primary endpoint of 6-minute walk distance between the treatment and control groups was statistically significant, it did not appear to be clinically meaningful ^69^.

Bronchoscopic lung volume reduction with the use of one-way endobronchial valves is another potential treatment for patients with severe emphysema. To date, the benefits have been modest but have been hypothesized to be much larger in patients without interlobar collateral ventilation than in those with collateral ventilation. A single-center, double-blind, sham-controlled trial in patients with severe COPD, significant hyperinflation, and restricted exercise tolerance with a target lobe with intact interlobar fissures on chest computed tomography showed significant improvement in lung function at 3 months ^70^. A subsequent study of 64 patients randomly assigned to the endobronchial valve group or the control group and intention-to-treat analyses showed greater improvements in the pulmonary function and exercise capacity in those treated with endobronchial valves ^71^. Further investigation is warranted to determine whether this will be a potential approved therapy in patients with severe COPD and intact fissures.

A characteristic of COPD is a disproportionately high prevalence of common comorbidities such as cardiovascular disease, diabetes, lung cancer, depression, metabolic syndrome, skeletal muscle dysfunction, and osteoporosis ^14^. These comorbidities are so common that they are now part of the GOLD definition ^14^ and can occur in patients with mild, moderate, or severe airflow limitation ^72^. Comorbidities in COPD influence mortality and hospitalizations independently ^73^. COPD itself has significant systemic effects, including skeletal muscle dysfunction which may be characterized by loss of muscle cells or abnormal function of remaining cells or both ^74^. Although skeletal muscle dysfunction may be caused by inactivity, poor diet, inflammation, and hypoxia, it is a remediable source of exercise intolerance ^75^. Bronchoscopic therapies aim to widen the population to whom non-pharmacologic therapies are available. However, comorbidities may present challenges of candidacy and tolerance of procedures in addition to impacting the optimization of pulmonary rehabilitation after lung volume reduction procedures have been performed.

## Conclusions

Emerging evidence that withdrawal of ICS is safe in some patients makes combination LAMA/LABA pharmacotherapy a reasonable option for many, particularly those with persistent dyspnea on a single long-acting bronchodilator. The growth of novel dual-agent long-acting bronchodilator inhalers may decrease the excessive over-prescription of combination ICS/LABAs. Further evidence is needed to better understand the role of bronchoscopic lung volume reduction.
